# Preparation and bioactivities of selenium nanoparticles based on a polysaccharide from *Dendrobium huoshanense*

**DOI:** 10.3389/fnut.2025.1689214

**Published:** 2025-12-10

**Authors:** Qingyan Pei, Leilei Zhang, Yupeng Yang, Changchang Gao, Bingji Ma, Yirong Xi, Li Wang

**Affiliations:** 1 College of the Fifth Clinical Medical (Zhengzhou People's Hospital), Henan University of Chinese Medicine, Zhengzhou, China; 2 Guochen Technology (Henan) Group Co., Ltd., Zhengzhou, China; 3 Department of Traditional Chinese Medicine, College of Agronomy, Henan Agricultural University, Zhengzhou, China

**Keywords:** *Dendrobium huoshanense* polysaccharide, structural characterization, polysaccharide selenium nanoparticles, antioxidant activities, immune activities

## Abstract

The present work reported the preparation and characterization of a polysaccharide (DPHs-1) from *Dendrobium huoshanense* and its influence on bioactivities of selenium nanoparticles (DPH-SeNPs). DPH-SeNPs were synthesized using DPHs-1 as stabilizer and dispersant. Experiments were conducted to investigate the effects of DHPs-1 concentration, temperature, the molar ratio of Vitamin C (Vc) to Sodium selenite (Na_2_SeO_3_), and reaction time on the preparation of DHP-SeNPs. Scanning electron microscopy (SEM) revealed that the DHP-SeNPs had a relatively rough and uneven surface. Transmission electron microscopy (TEM) imaging showed DHP-SeNPs were sphere-like in morphology and homogeneously distributed. X-ray diffraction (XRD) analysis indicated that the diffraction peak range changed, leading to the formation of a new substance. The fourier transform infrared spectroscopy (FT-IR) indicated that the main interaction between DHPs-1 and SeNPs occurred in the Se-O bonds, further leading to the stable spherical structure of DHPs-1 decorated SeNPs. Congo red test results revealed that the triple-helix structure of DHP-SeNPs was intact. DHP-SeNPs displayed favorable thermal stability. *In vitro* antioxidant experiment results showed that DHP-SeNPs had strong scavenging abilities for the 1,1-diphenyl-2-picrylhydrazyl (DPPH), hydroxyl radical, and ABTS^+^ free radicals and they had good reducing power. The results of Cell Counting Kit-8 (CCK-8) experiments showed that both DHPs-1 and DHP-SeNPs with concentrations in the range of 5–320 μg/mL could promote cell proliferation without cytotoxicity. The results of scratch experiments showed that DHPs-1 and DHP-SeNPs, within concentrations of 20, 40, and 80 μg/mL, significantly promoted scratch healing of macrophage RAW264.7. Bioactivity tests indicated that DHPs-1 and DHP-SeNPs promoted the release of tumor necrosis factor-*α* (TNF-α), interleukin-1β (IL-1β), interleukin-6 (IL-6), and interleukin-10 (IL-10) through upregulating the mRNA expression of TNF-α, IL-1, IL-6, and IL-10. In conclusion, DPH-SeNPs have novel bioactivities with promising applications in food and biomedicine.

## 1 Introduction

*Dendrobium huoshanense*, a valuable traditional Chinese medicinal herb, boasts a medicinal history exceeding a thousand years in China and is ranked first among the top 10 medicinal materials of Anhui Province (1). The polysaccharide components in *D. huoshanense* are among its most representative active substances. The elements of selenium are essential for many anti-inflammatory, antibacterial and antioxidant activities and is very important for human growth and development (2, 3). Moreover, glutathione peroxidase which contains selenium, protects against oxidative stress damage caused by free-radicals (4). However, selenium cannot be synthesized by the human body and must be sourced externally, primarily in its inorganic form (5). Inorganic selenium exhibits low bioavailability and potential toxicity. Poisoning occurs when selenium absorption exceeds safe limits, significantly restricting its use (6). In recent years, selenium nanoparticles (SeNPs) have attracted considerable attention due to their advantages of higher bioactivity and lower toxicity (7). Nanoparticles themselves also possess unique functional properties, including antioxidant and immunomodulatory effects (8, 9). Meanwhile, polymers are easy to modify and can form structures with novel and improved activities, laying a foundation for the development of functional materials (10–13). Polysaccharides are widely present in organisms as natural biomacromolecules, with good biocompatibility, polysaccharides can also bind to specific cell receptors in a targeted manner to exert their unique biological activities (14). Based on this, researchers have combined polysaccharides with selenium to prepare nanoparticles with more excellent biological activity. For example, polysaccharides were isolated from *Codonopsis pilosula*, and polysaccharide-based selenium nanoparticles (CPW1-Se) were successfully prepared—this material showed significant effects in regulating cell proliferation and apoptotic activity (15), *Lycium barbarum* polysaccharide-based selenium nanoparticles can effectively alleviate fatigue by increasing glycogen reserves, enhancing the level of antioxidant enzymes, and regulating metabolic mechanisms (16).

In our preliminary studies, we successfully isolated the polysaccharide DHPs-1 from *Dendrobium huoshanense* and verified its antioxidant and immunomodulatory activities (17). In this study, DHPs-1 was combined with SeNPs to prepare the composite DHP-SeNPs. This composite not only retains the advantages of both components but also exhibits enhanced antioxidant and immunomodulatory activities, providing a safe and efficient new approach for selenium supplementation in the human body and holding significant application prospects. The objective of this study is to synthesize DHP-SeNPs with stronger selenium-supplementing activity by modifying DHPs-1. In the experiment, DHPs-1 was used as a stabilizer and dispersant for SeNPs to prepare the target product DHP-SeNPs. The particle size of DHP-SeNPs was determined using a dual-wavelength method, and a laser diffraction particle size analyzer was employed to investigate the effects of various factors on the preparation conditions of DHP-SeNPs. Meanwhile, in-depth studies were conducted on the antioxidant and immunomodulatory activities of DHP-SeNPs. DHP-SeNPs has been successfully synthesized. Through single-factor experiments, the effects of different preparation conditions on the DHP-SeNPs products were explored, aiming to improve their stability and preparation efficiency, enhance their bioactivity, and provide theoretical support for their applications in biomedicine and food industry.

## 2 Materials and methods

### 2.1 Materials and reagents

The *D. huoshanense* is sourced from Huoshan County, Anhui Province. DPPH (1,2-Diphenyl-2-picrylhydrazyl, Tokyo Chemical Industry Co., Ltd., Japan), CCK-8 (Hanheng Biotechnology Co., Ltd.), dimethyl sulfoxide, RPMI1640 medium (Thermo Fisher Scientific Biochemical Products Co., Ltd.), fetal bovine serum (Tianhang Biotechnology Co., Ltd.), lipopolysaccharide (LPS, Sigma-Aldrich Trading Co., Ltd.), trypsin–EDTA, penicillin/streptomycin solution, cell lysis buffer, neutral red (Beyotime Biotechnology Co., Ltd., Shanghai), Trizol (Invitrogen Life Technologies Co., Ltd., United States), 4 × gDNA wiper Mix (Anqing Techron Piston Ring Co., Ltd.), all other chemicals or reagents were of analytical grade. CO_2_ Incubator (Thermo Fisher Scientific Biochemical Products Co., Ltd.), BioTek Epoch Full Wavelength Microplate Reader (Thermo Fisher Scientific Biochemical Products Co., Ltd.), Inverted Biological Microscope (Olympus Corporation, Japan), Life Technologies 7,500 Fast Fluorescence Quantitative PCR Instrument (Thermo Fisher Scientific Instrument Co., Ltd.), Benchtop High-Speed Centrifuge (Eppendorf AG, Germany), Discovery DV215CD Precision Electronic Balance (OHAUS Corporation, United States), LS13320 Laser Diffraction Particle Size Analyzer (Beckman, United States).

### 2.2 Preparation of DPH-SeNPs

**Figure 1:**
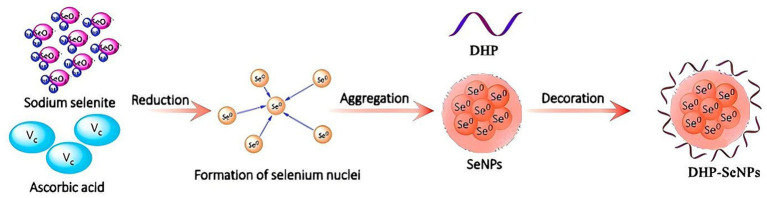
Schematic diagrams of formation mechanism of DHP-SeNPs.

According to our previous studies, *D. huoshanense* polysaccharide (DHPs-1) were prepared (17). The synthesis of DPH-SeNPs was carried out using a modified version of the method from Zhou et al. (18). A specific amount of DHPs-1 was accurately weighed into a conical flask, and a predetermined volume of sodium selenite solution was added. The mixture was stirred using a magnetic stirrer for 1 h. Different concentrations of ascorbic acid were then gradually added dropwise. The mixture was heated at varying temperatures for different durations and subsequently dialyzed using a 3,500 Da dialysis bag to obtain the DHP-SeNPs solution. The schematic diagrams of the DHP-SeNPs production is shown in Figure 1.

### 2.3 Dual-wavelength method for particle size determination

Based on the ual-wavelength principle of colloidal solutions, solutions of DHPs-1, Vc + sodium selenite, Vc + DHPs-1, Vc, and DHPs-1 were prepared at appropriate concentrations for size determination using the dual-wavelength absorbance ratio method. The absorption spectra of these solutions were determined, and plots were constructed with absorbance values (or A_2_/A_1_ ratios) as the y-axis and various evaluation factors as the x-axis to characterize the size of SeNPs.

### 2.4 Laser diffraction particle size analysis

Particle size of the prepared DHP-SeNPs solution was determined using the LS13320 laser heating particle size analyzer. The analysis was conducted in triplicate, with 3 to 5 parallel measurements performed at an environmental temperature of 25 °C, and the data were subsequently recorded.

### 2.5 Single factor testing of synthesis conditions

#### 2.5.1 DHPs-1 concentration

Different concentrations of DHPs-1 (0, 0.2, 0.4, 0.6, 0.8 and 1.0 mg/mL) was used in combination with 1 mM Na_2_SO_3_ solution and stirred on a heated magnetic stirrer for 1 h. The Na_2_SO_3_ solution was then added to the same amount of 3 mM Vc solution. The temperature was held at 40 °C for 4 h, after which a specific volume of the reaction mixture was then taken, and the absorbance at wavelengths of 410 nm and 490 nm was measured.

#### 2.5.2 Reaction temperature

0.2 mg/mL of DHPs-1 was mixed with a 1 mM Na_2_SeO_3_ solution and stirred thoroughly for 1 h using a heated magnetic stirrer to ensure complete mixing. Subsequently, add an equal volume of a 3 mM Vc solution was added to the Na_2_SeO_3_ solution. Reactions were carried out at 30, 40, 50, 60, and 70 °C for 4 h. Afterward, a specified volume of the reaction solution was removed, and absorption was measured at wavelengths of 410 and 490 nm.

#### 2.5.3 Vc: Na_2_SeO_3_ ratio

0.2 mg/mL of DHPs-1 was mixed with a 1 mM Na_2_SeO_3_ solution, and stirred on a heated magnetic stirrer for 1 h. Subsequently, Vc was added in volumes equal to that of the Na_2_SeO_3_ solution, at varying concentrations (1, 2, 3, 4, 5, and 6 mM), and the reaction was allowed to proceed at 40 °C for 4 h. Afterward, a specified volume of the reaction solution was removed, and absorption was measured at wavelengths of 410 and 490 nm.

#### 2.5.4 Reaction time

0.2 mg/mL of DHPs-1 was mixed with 1 mM of Na_2_SeO_3_ solution and stirred with a heated magnetic stirrer for 1 h. The same volume of 3 mM Vc solution as the Na_2_SeO_3_ solution was added. The temperature was maintained at 40 °C as the reaction was allowed to proceed for different time periods (1, 2, 3, 4, 5, 6 h). A specific volume of the reaction solution was removed, and absorption was measured at wavelengths of 410 and 490 nm.

### 2.6 Effect of temperature and Vc: Na_2_SeO_3_ ratio by laser diffraction particle size analyzer

Based on the results of the 2.5 experiment, the reaction temperature and the ratio of ascorbic acid to sodium selenite significantly influence the synthesis process. To further confirm this, the particle size of the prepared particles was analyzed using a laser diffraction particle size analyzer, following the methods outlined in sections 2.5.2 and 2.5.3.

### 2.7 Determination of selenium content

The SeNPs solution was diluted to a specific concentration, and 10 mL of this solution was taken. Then 20 mL of concentrated nitric acid was added. The mixture was covered with watch glass, and allowed to cool at room temperature overnight. The solution was then heated on a hot plate until it became clear and colorless. After cooling, 10 mL of concentrated hydrochloric acid was added, and heating continued until the solution was again clear and colorless. After allowing the solution to cool, dilute it with distilled water until the total volume reaches 100 mL in a volumetric flask. Following these steps, a standard selenium curve was constructed from which the absorptions of the prepared sample solutions were determined. To calculate the selenium content in each sample, the obtained absorption value was inserted into the standard curve equation.

### 2.8 Structural characterization and analysis

#### 2.8.1 Scanning electron microscopy analysis

After coating the sample with a gold film using a vacuum sputtering coater, it was analyzed via SEM employing the Quanta 250 model from FEI, United States, with an acceleration voltage set to 5 kV.

#### 2.8.2 Transmission electron microscopy analysis

The sample was placed on a dry copper grid. Subsequently, observations were conducted using the Tecnai G2 Spirit Bio transmission electron microscope (FEI, Czech Republic).

#### 2.8.3 X-ray diffraction analysis

The samples were ground into a fine powder and placed on a sample holder. The crystal structure of the samples was analyzed using an X-ray diffractometer (Mini Flex 600, Japan) at a temperature of 25 °C, within a 2θ range of 5° to 80°.

#### 2.8.4 Infrared spectroscopy analysis

The appropriate amount of the sample was thoroughly dried and subsequently ground with anhydrous potassium bromide to form a pellet. Infrared spectroscopic analysis was conducted using a Bruker Vector 22 spectrometer, scanning over the wavenumber range of 4,000 to 400 cm^−1^.

#### 2.8.5 Thermogravimetric analysis

Approximately 5 mg of the sample was placed in a sealed Tzero aluminum pan and measured in dynamic nitrogen. The temperature range was 25–800°, and the rate of increase was 10°/min. Thermal analysis was carried out with a thermogravimetric analyzer from Shimadzu, Japan.

#### 2.8.6 Congo red test

Prepared solutions consisted of 100 mL of a 100 μM Congo red solution and a 1 M NaOH solution. The sample solution, totaling 1 mL, was added to 1 mL of the Congo red solution. Afterward, the 1 M NaOH solution was slowly introduced to reach final concentrations of 0, 0.05, 0.1, 0.15, 0.2, 0.25, 0.3, 0.35, 0.4, 0.45, and 0.5 mol/L. The mixtures were left at room temperature for 10 mins before measuring the peak absorption wavelength between 400 and 600 nm using a UV–Vis spectrophotometer at different concentrations (19).

### 2.9 Antioxidant studies

#### 2.9.1 Determination of reduction ability

A pipette was used to transfer 200 μL of sample solutions with concentrations ranging from 0.05 to 10 mg/mL into test tubes. Each tube received an addition of phosphate buffer solution and potassium ferricyanide solution. The solutions were maintained at 50 °C for 20 min and subsequently cooled to room temperature. Afterward, trichloroacetic acid and FeCl_3_ solution were introduced, and the mixtures were mixed thoroughly. Using Vc as the positive control, the absorbance at 700 nm was measured. A higher absorbance value indicated a stronger reducing capacity of the polysaccharide samples.

#### 2.9.2 Hydroxyl radical scavenging assay

The capacity of hydroxyl radical scavenging was gently assessed following the approach by Yao (20). An equal volume of FeSO_4_ solution and salicylic acid ethanol solution was added to 50 μL of samples at different concentrations. After adding H_2_O_2_, the mixture was kept at 37 °C for 30 min. Using Vc as a positive control, the absorbance was determined at 510 nm. This formula was used to calculate the hydroxyl radical scavenging rate of the polysaccharide samples:


$$\text{Hydroxyl radical scavenging rate}\;(\%)=[1-\frac{A_{X}-A_{X0}}{A_{0}}]\times 100$$

Where A_0_ is the absorbance of the blank, A_X_ is the absorbance of the sample solution, and A_X0_ is the absorbance when distilled water replaces the H_2_O_2_ solution.

#### 2.9.3 DPPH radical scavenging rate assay

The sample solution was mixed with the DPPH solution, and the absorbance was measured at 517 nm after the reaction, using Vc as the positive control (21). The formula to determine the DPPH free radical scavenging rate is as follows:


$$\text{DPPH radical scavenging rate}\;(\%)=[1-\frac{A_{X}-A_{X0}}{A_{0}}]\times 100$$

Where A_0_ is the absorbance of the solution without the sample mixture, A_X_ is the absorbance of the sample solution group, and A_X0_ is the absorbance of the mixture without DPPH solution.

#### 2.9.4 ABTS^+^ radical scavenging capacity assay

The ability to scavenge ABTS^+^ radicals was assessed using a slightly modified version of the method from Jia (22). ABTS reagent was mixed with a K_2_S_2_O_8_ solution and then incubated in darkness for 12 to 16 h. The solution was subsequently diluted with phosphate-buffered saline (PBS) until it reached an absorbance of 0.70 ± 0.05. The samples were mixed with the ABTS solution, with Vc serving as the positive control (23). The rate at which ABTS^+^ radicals are scavenged was determined using the following formula.


$$\text{ABTS}+\text{radical scavenging capacity}(\%)=[\frac{A_{0}-(A_{X}-A_{X0})}{A_{0}}]\times 100$$

Where A_0_ is the absorbance of the distilled water and ABTS mixed solution, A_X_ is the absorbance of the polysaccharide sample solution mixed with ABTS reagent, and A_X0_ is the absorbance of polysaccharide sample solution in PBS buffer.

### 2.10 Immunomodulatory activity studies

#### 2.10.1 Cell culture

The RAW264.7 cell line was obtained from the Shanghai Institute of Biological Sciences, Chinese Academy of Sciences. These cells were cultured in RPMI 1640 medium supplemented with 10% fetal bovine serum and 1% streptomycin/penicillin, maintained at 37 °C in an incubator with 5% CO_2_ (24).

#### 2.10.2 Cell viability assay

The cell viability was evaluated using a method adapted from Yang (24). For 24 h, RAW264.7 cells were cultured in a 96-well plate, after which they were treated with varying concentrations of DHPs-1 and lipopolysaccharide (LPS) as a positive control (1 μg/mL) for an incubated 24 h. Afterward, 10 μL of CCK-8 reagent was added to each well, followed by a one-hour incubation at 37 °C before measuring absorbance at 450 nm. Calculations were performed according to following formula.


$$\text{Cell Viability Assay}(\%)=[\frac{{\mathrm{OD}}_{\text{DHPs}-1}-{\mathrm{OD}}_{\text{Blank}}}{{\mathrm{OD}}_{\text{Control}}-{\mathrm{OD}}_{\text{Blank}}}]\times 100$$

#### 2.10.3 Phagocytic capacity assay

The phagocytic capacity was evaluated using a method adapted from Yang (24). RAW264.7 cells were placed at a concentration of 2 × 10^4^ cells per well in a 96-well plate and incubated overnight at 37 °C with 5% CO_2_. After the incubation period, the supernatant was removed, and the cells were washed three times with PBS. Subsequently, a solution of 0.1% neutral red staining dye was added, and the cells were incubated for an additional 4 h. Afterward, PBS was used to wash away any unphagocytosed neutral red dye. Add the lysis buffer, incubate at room temperature for 4 h, and subsequently measure the absorbance at 540 nm. The following formula was used to compute the phagocytic index:


$$\text{Phagocytic Index}(\%)=[\frac{\text{experimental group}\;\mathrm{OD}}{\text{blank control group}\;\mathrm{OD}}]\times 100$$

#### 2.10.4 Cell scratch assay

A density of 1 × 10^6^ RAW264.7 cells was used to seed each well of six-well plates. After a 24-h incubation, straight lines were drawn in each well of the culture plate, afterwards, PBS was used to wash away the cells that had become detached. Subsequently, different concentrations of samples and LPS-containing medium were added and the plates were incubated for another 24 h. The cell migration was recorded, and the following formula was used to calculate the cell migration rate:


$$\text{Cell Migration Rate}(\%)=[\frac{H_{1}-H_{2}}{H_{1}}]\times 100$$

Where H_1_ is the average width of scratch healing at 0 h, and H_2_ is the average width of scratch healing at 24 h.

#### 2.10.5 RT-PCR assay

The mRNA expression levels of TNF-*α*, IL-1β, IL-6, and IL-10 were determined using a modified approach based on the methodology described by Luo (25). The mRNA transcription levels of TNF-*α*, IL-1β, IL-6, and IL-10 were analyzed. Specifically, RAW264.7 cells were cultured with the tested samples (20, 40, and 80 μg/mL) at 37 °C for 24 h in the presence of LPS (1 μg/mL) as a standard. Glyceraldehyde-3-phosphate dehydrogenase (GAPDH) was used as an internal control to quantify the expression levels of TNF-α, IL-6, IL-10, and IL-1β mRNA. The primer design for these four cytokines is detailed in Table 1.

**Table 1:** Sequences and amplification conditions for RT-PCR primers.

Primer	Sequences	Annealing temperature (°C)
GAPDH	F5′-ACCCCAGCAAGGACACTGAGCAAG-3′ R5′-GGCCCCTCCTGTTATTATGGGGGT-3′	60
TNF-a	F5′-GGAAAGGACACCATGAGC -3′ R5′-CCACGATCAGGAAGGAGA-3′	64
IL-1β	F5′-CCGCAGCCTACATCATTC-3′ R5′- CGCCATAAGCCCTCCTA −3′	63
IL-6	F5′- TGTGTGAAAGCAGCAAAGA −3′ R5′- ACCAGGCAAGTCTCCTCA −3′	62.5
IL-10	F5′- GCCCTTTGCTATGGTGTC -3′ R5′- TCTCCCTGGTTTCTCTTCC -3′	63.1

### 2.11 Statistical analysis

Graph processing was conducted through Origin 2024. Statistical significance was measured via Duncan’s Multiple Range Test through one-way ANOVA, with *p* < 0.05 holding statistical significance. Our measurements were conducted in triplicate, with all data denoted as mean ± standard deviation (n ≥ 3).

## 3 Results and discussion

### 3.1 DHP-SeNPs preparation

#### 3.1.1 Dual-wavelength method evaluation

**Figure 2:**
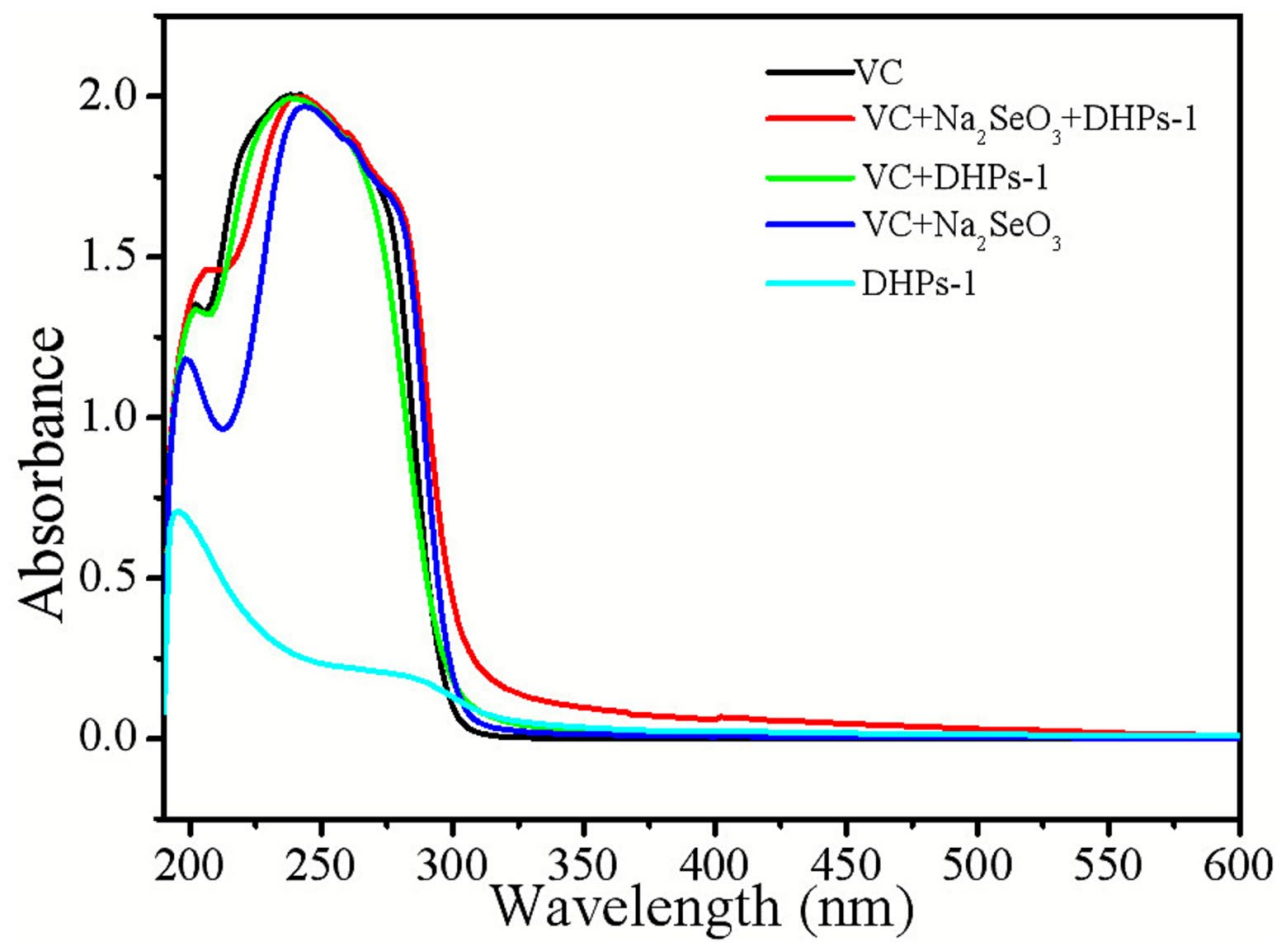
UV-absorption spectra of different solutions.

As illustrated in Figure 2, the DHPs-1 solution exhibits a maximum absorption peak at 196 nm. The Vc solution exhibits a maximum absorption at 238 nm, while the mixed solution of Vc and Na_2_SeO_3_ reveals a red-shift with the absorption peak reaching its maximum at 244 nm. The DHPs-1 + Vc + Na_2_SeO_3_ mixture has a maximum absorption peak at 242 nm. Notably, aside from the DHPs-1 + Vc + Na_2_SeO_3_ combination, other solutions exhibit minimal ultraviolet absorption within the range of 300 to 600 nm. This indicates that the UV absorption spectrum of the synthesized DHP-SeNPs is fundamentally distinct from other mixed solutions, suggesting that the DHP-SeNPs are a new substance.

According to the dual-wavelength method, a constant absorbance ratio A_2_/A_1_ implies that the colloidal particles are in a stable state with unchanged particle size. Furthermore, an increasing ratio correlates with smaller particle sizes and more stable morphologies for the synthesized selenium nanoparticles. To avoid interference from Vc absorbance and facilitate measurement, wavelengths of 410 nm and 490 nm in the visible region were selected for determining nanoparticle selenium solutions. The absorbance ratio A_410_/A_490_ was employed for characterizing changes in particle size of DHP-SeNPs products (26).

#### 3.1.2 Evaluation of the synthesis conditions for DHP-SeNPs

##### 3.1.2.1 Effect of DHPs-1 concentration on the A_410_/A_490_ ratio

**Figure 3:**
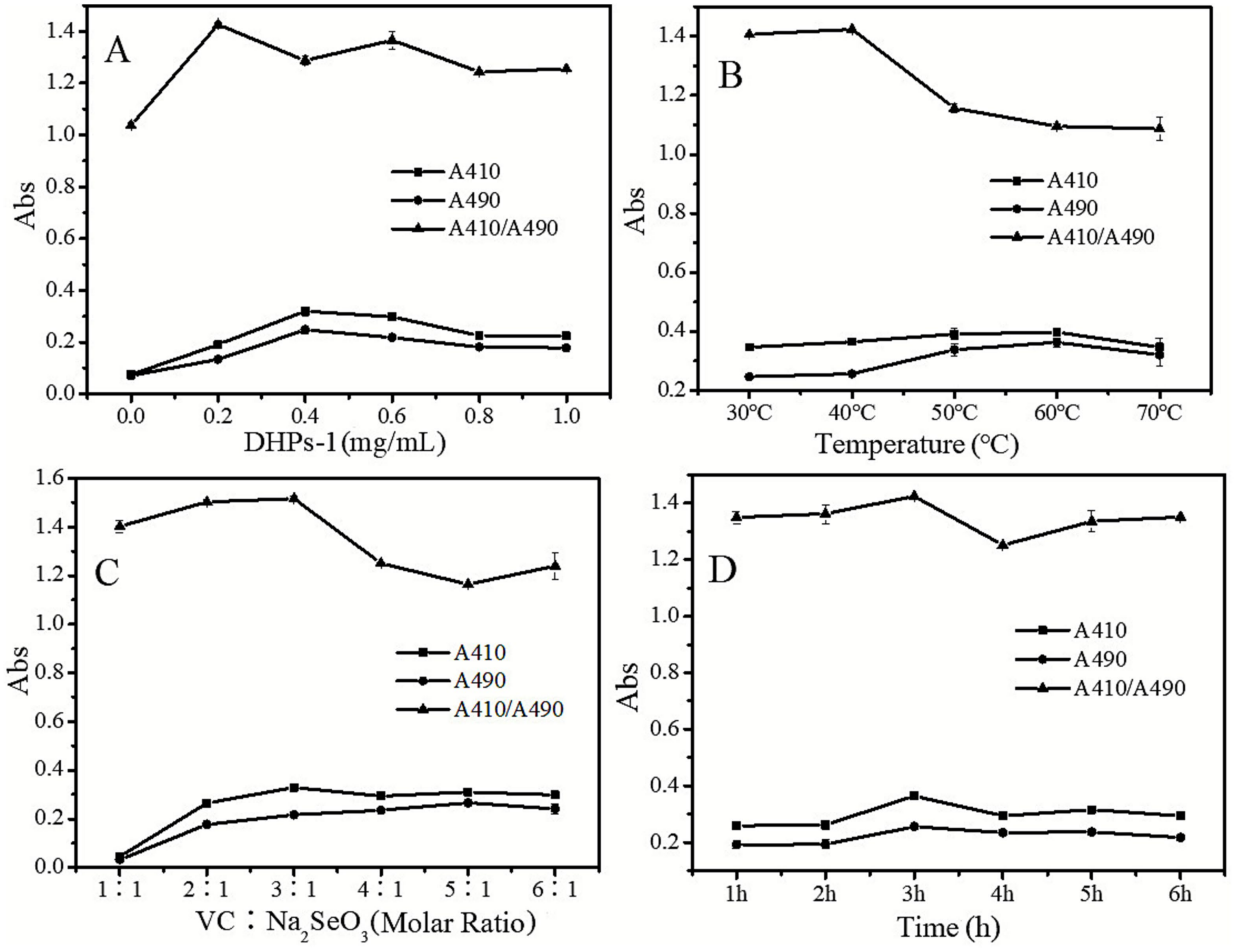
**(A)** Effect of DHPs-1 concentration on *A_410_/A_490_*; **(B)** Effect of reaction temperature on *A_410_/A_490_*; **(C)** Effect of ratio of Vc and sodium selenite on *A_410_/A_490_*; **(D)** Effect of reaction time on *A_410_/A_490_*.

As illustrated in Figure 3A, when the amount of polysaccharide varies from 0.2 to 1 mg/mL, the A_410_/A_490_ ratio remains stable within the range of 1.2 to 1.4, which is consistently higher than the ratio observed at 0 mg/mL polysaccharide concentration. This result indicates that, in the absence of DHPs-1, the generated SeNPs is highly unstable due to the lack of stabilizing effects from polysaccharides, leading to aggregation and an increase in particle size. Experimental observations revealed that the without DHPs-1 produced dark red SeNPs, which rapidly formed precipitates. In contrast, the system with DHPs-1 exhibited a bright red color and remained stable without precipitation. This discrepancy may be attributed to the fact that, following the reduction of sodium selenite by Vc, elemental selenium tends to aggregate. However, the presence of DHPs-1 in the solution effectively prevents further aggregation of elemental selenium through its hydroxyl groups and hydrogen bonding interactions, thereby controlling the particle size of SeNPs. Therefore, by changing the levels of DHPs-1, the growth of SeNPs can be regulated to achieve SeNPs of different sizes. Based on Figure 3A, it was determined that a DHPs-1 concentration of 0. 2 mg/mL is suitable conditions for the preparation DHP-SeNPs.

##### 3.1.2.2 Influence of reaction temperature on the A_410_/A_490_ ratio

As shown in Figure 3B, at a reaction temperature of 40 °C, the A_410_/A_490_ ratio reaches its maximum. According to the dual-wavelength method analysis, under this condition, the generated SeNPs particles were smaller size and more stable. When the temperature increases from 50 °C to 70 °C, there is a sharp decline in the A_410_/A_490_ ratio, indicating that SeNPs struggle to maintain stability at high-temperatures. This observation aligns with our previous research (17). This phenomenon may be attributed to increased molecular motion within the solution due to rising temperatures, which facilitates closer contact among elemental selenium particles and promotes agglomeration into larger-sized aggregates. Based on Figure 3B, an suitable preparation temperature for DHP-SeNPs is determined as 40 °C.

##### 3.1.2.3 Influence of the molar ratio of Na_2_SeO_3_ on the A_410_/A_490_ absorbance ratio

As shown in Figure 3C, with the increase of the molar ratio of Vc to Na_2_SeO_3_, the A_410_/A_490_ ratio initially rises and then declines. When the molar ratio of Vc to Na_2_SeO_3_ reaches 3:1, the A_410_/A_490_ ratio attains its peak value, indicating that under these conditions, DHP-SeNPs exhibit stability, minimal particle size, and uniform distribution. Theoretically, the redox stoichiometric relationship between Vc and Na_2_SeO_3_ should be 2:1. However, experimental results suggest that a slight excess of Vc contributes to maintaining stability within the reaction system. In summary, for synthesizing stable and uniformly distributed DHP-SeNPs with smaller particle sizes, an suitable molar ratio of Vc to Na_2_SeO_3_ is 3:1.

##### 3.1.2.4 Impact of reaction time on the A_410_/A_490_ ratio

As illustrated in Figure 3D, the effect of reaction time on the A_410_/A_490_ ratio is not significant. However, this ratio reaches its maximum at 3 h. As Vc reduces Na_2_SeO_3_, the color of the solution gradually transitions from colorless and transparent to light yellow, then orange, and ultimately to a vivid red. This process reflects the phenomenon of increasing particle size due to agglomeration of elemental selenium. If the reaction time is prolonged, excessive agglomeration of elemental selenium occurs, resulting in an increase in particle size, consequently, a decrease in the A_410_/A_490_ ratio.

#### 3.1.3 Influence of reaction temperatures and Vc: Na_2_SeO_3_ molar ratios on the particle size of DHP-SeNPs

**Figure 4:**
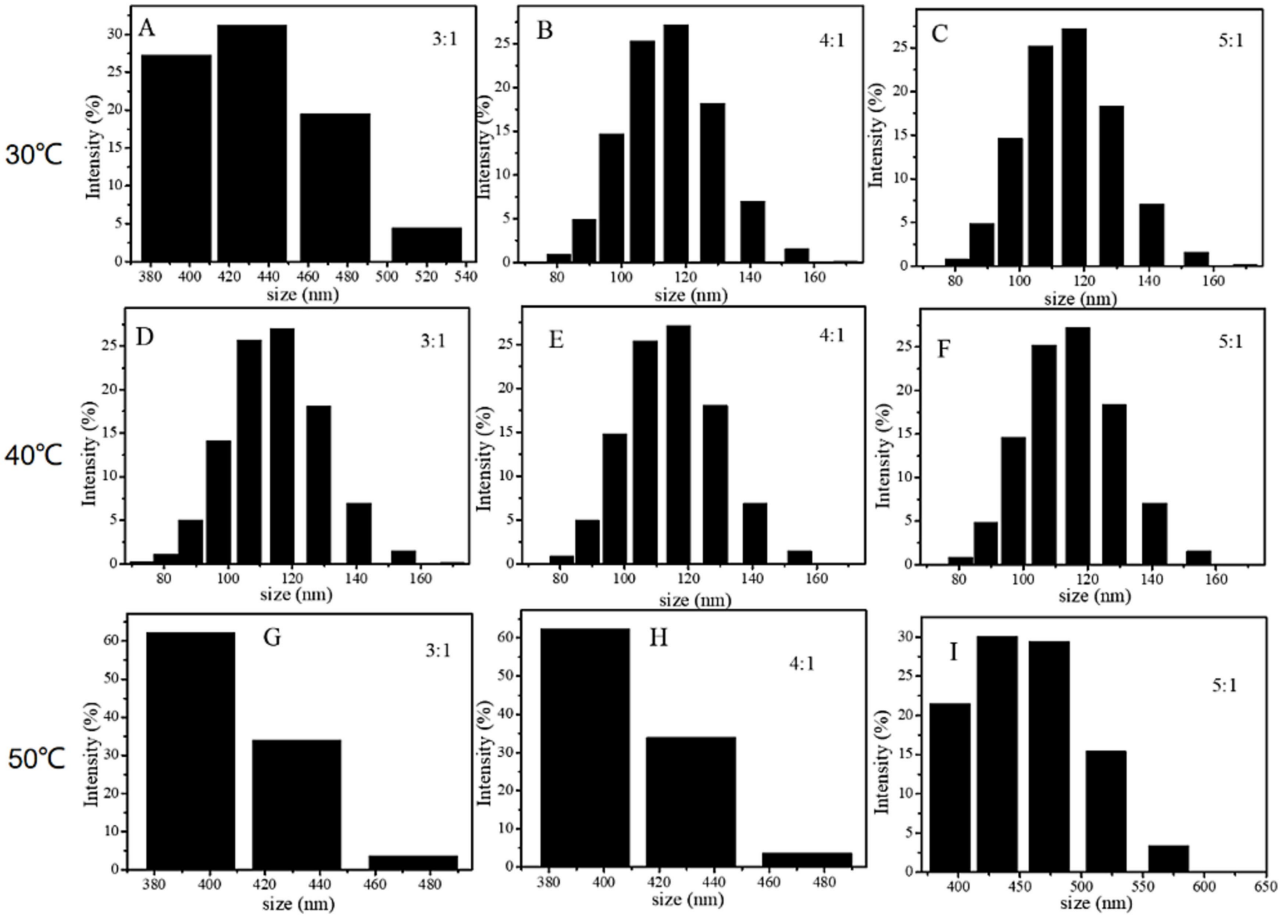
Effect on the size of DHP-SeNPs of reaction temperature and ratio of Vc: Na_2_SeO_3_. **(A)** 30 °C, 3:1; **(B)** 30 °C, 4:1; **(C)** 30 °C, 5:1; **(D)** 40 °C, 3:1; **(E)** 40 °C, 4:1; **(F)** 40 °C, 5:1; **(G)** 50 °C, 3:1; **(H)** 50 °C, 4:1; **(I)** 50 °C, 5:1.

In the preparation of DHP-SeNPs, Vc serves dual functions as a reducing agent and a stabilizer. Its core role is as a reducing agent, which can reduce Se^4+^ in the raw material Na₂SeO₃ to Se^0^ — the form that enables nanoparticle formation (27). The particle size distribution of nano-selenium at varying temperatures and different molar ratios of Vc: Na_2_SeO_3_ was assessed using a laser diffraction particle size analyzer. As illustrated in Figure 4, the average particle sizes are as follows: At 30 °C with Vc: Na_2_SeO_3_ ratios of 3:1, 4.1 and 5:1, the average particle sizes are 359.18 nm, 114.01 nm, and 114.12 nm, respectively. At 40 °C (3:1 to 5:1), the average particle sizes measure 113.87 nm, 113.89 nm, and 114.13 nm, respectively. At 50 °C (3:1 to 5:1), the recorded average particle sizes are 409.17 nm, 409.13 nm, and 454.39 nm, again, respectively.

As illustrated in Figure 4A, these average particle sizes are significantly higher than those observed under equivalent temperature conditions in Figures 4B,C. This discrepancy may be attributed to lower molecular motion rates at reduced temperatures, which hindered the adsorption of elemental selenium onto polysaccharide surfaces after reduction, resulting in larger nanoparticle formation.

According to Figures 4B,C, when the temperature is 30 °C, increasing the concentration of Vc causes the particle size to reduce. This occurrence might be due to the fact that at elevated concentrations of Vc, selenite ions (SeO_3_^2−^) was reduced more rapidly, resulting in a greater production of SeNPs that are more readily adsorbed onto the surface of polysaccharides, thereby leading to smaller particle sizes.

From Figures 4D–F, it can be inferred that at 40 °C, different molar ratios of Vc: Na_2_SeO_3_ yield similar nanoparticle sizes and comparable size distributions for SeNPs. This indicates that reaction temperature significantly influences the particle size during DHPs-1 formation. An excess amount of Vc contributes to maintaining stability within the reaction system. However, when its ratio exceeds 3:1, its effect on particle size becomes minimal. The particle size distribution is mainly between 70 nm and 170 nm at a temperature of 40 °C, with a fairly consistent distribution. At 50 °C, in contrast, the average size of the particles formed is greater than 400 nm. This observation suggests that SeNPs produced under these conditions exhibit instability. Nanose selenium particles are prone to aggregation under these conditions, significantly increasing the particle size of nanose selenium. Due to the scale effect associated with selenium nanoparticles, smaller particle sizes correlate with higher biological activity. Smaller SeNPs can effectively scavenge free radicals both *in vivo* and *in vitro*, exhibiting various biological activities (28). Based on the measurement results obtained from dual-wavelength methods and laser particle size analyzers, as well as adherence to the principle of material conservation, we selected a polysaccharide concentration of 0.2 mg/mL, a reaction temperature of 40 °C, a molar ratio of Vc: Na_2_SeO_3_ at 3:1, and a reaction time of 3 h as the suitable conditions for preparing DHP-SeNPs.

#### 3.1.4 Physicochemical properties of DHP-SeNPs

According to Table 2, the total sugar content of DHP-SeNPs is 83.56%. The observed decrease in sugar content can be attributed to the adsorption of SeNPs onto the surface of DHPs-1, which results in an increased selenium content and a relative reduction in total sugar levels. The standard curve for selenium content measured by method 2.5.6 is represented as: Y = 1.0691x + 0.002 (R^2^ = 0.9991). The selenium content in dried DHP-SeNPs is determined to be 11.15%. The protein content increased from 2.7 to 3.76%, which may be attributed to the binding of amino acids in the protein with SeNPs, resulting in a relative increase in content. Additionally, a small amount of polyphenols was detected in the samples. X-ray diffraction analysis revealed that the crystallinity of DHP-SeNPs was 13.96%, lower than that of DHPs-1, indicating a decrease in crystallinity and suggesting that DHP-SeNPs may possess improved solubility. Although there was a reduction in molecular weight, the change in dispersity index was minimal, indicating that the formation of SeNPs through the combination of SeNPs and DHPs-1 did not alter the homogeneity of DHPs-1.

**Table 2:** Physicochemical properties of DHP-SeNPs.

Physicochemical properties	DHP-SeNPs
Total sugar content (%)	83.56 ± 0.93
Protein content (%)	3.76 ± 0.05
Polyphenol content (%)	0.66 ± 0.01
Crystallinity (%)	13.96 ± 0.15
Selenium content (%)	11.15 ± 0.30
Molecular weight (KDa)	
Weight-average molecular weight (Mw)	37.17 ± 3.547
Number-average molecular weight (Mn)	27.93 ± 1.78
Polydispersity index (PDI)	1.331 ± 3.97

#### 3.1.5 SEM

**Figure 5:**
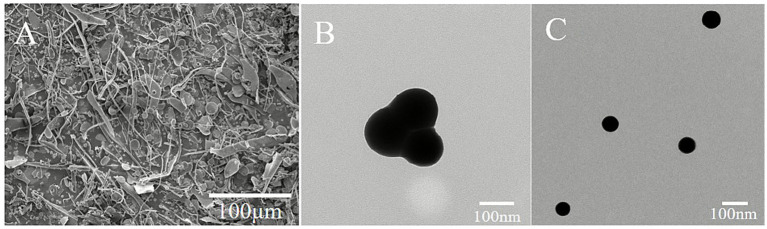
SEM images of **(A)** DHP-SeNPs; TEM images of **(B)** SeNPs and **(C)** DHP-SeNPs.

The surface of DHPs-1, as depicted in Figure 5A, appears smooth and ribbon-shaped. After the adsorption of SeNPs onto DHPs-1, the surface morphology of DHP-SeNPs transforms into a rough and uneven particulate structure. This change may be attributed to the adsorption of SeNPs on the polysaccharide surface, which alters the originally smooth surface of DHPs-1 into a granular form. This indicates that the formation of DHP-SeNPs modifies the surface morphology of DHPs-1. The similar result was obtained by Sun et al. (28), in their study on preparing nano-selenium using hawthorn polysaccharides through SEM observation, thereby further validating the impact of SeNPs on polysaccharide surface morphology.

#### 3.1.6 TEM

TEM is an effective observational method for analyzing the surface morphology and particle size of nanoparticles (29). As illustrated in Figures 5B,C, the morphology and particle size of SeNPs generated from a reaction system without DHPs-1, as well as those produced with the addition of DHPs-1 to form DHP-SeNPs, were examined through TEM. According to the results, DHP-SeNPs exhibited a monodisperse, uniformly spherical morphology with an average particle size of 61 nm smaller than polysaccharide-derived SeNPs such as those from goji berries (105.4 nm) (30), dragon beard kelp polysaccharide (92.5 nm) (31), and green tea polysaccharide (70 nm) (32). This smaller particle size implies enhanced stability, suggesting that DHPs-1 effectively promoted the dispersion and stabilization of SeNPs, thereby facilitating the formation of more stable selenium nanoparticles.

The particle size of SeNPs, as shown in Figure 5B, reaches about 260 nm, which is approximately four times that of DHP-SeNPs. Additionally, the morphology of SeNPs is irregular and non-uniform. The particle size analysis results obtained from the laser diffraction particle size analyzer indicated that the average particle size of DHP-SeNPs under specifc conditions was about 113 nm, which is significantly larger than that observed via TEM. This discrepancy is probably due to the fact that TEM primarily focuses on the SeNPs themselves, while the laser diffraction particle size analyzer measures the overall size of the DHP-SeNPs nanocomposite, which comprises both DHPs-1 and SeNPs (30). TEM revealed their spherical shape. The spherical morphology may represent a more favorable configuration for the stability of polysaccharide selenium particles, as it is associated with lower surface energy, thereby facilitating their stable existence (31).

#### 3.1.7 XRD

**Figure 6:**
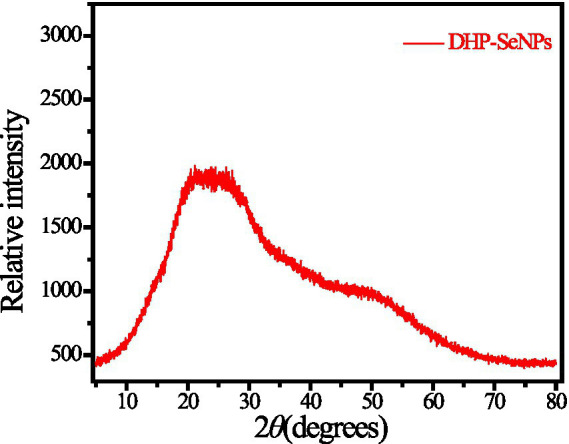
X-ray diffraction patterns of DHP-SeNPs.

Due to the unique diffraction patterns exhibited by different crystalline materials, XRD techniques can be employed to identify the crystal structures of nanoparticles. The broadening of XRD peaks confirms the formation of nanoscale particles. In cases where nanoparticles have an amorphous structure, no diffraction peaks will be observed. The smaller the nanoparticles, the broader the range of XRD peaks appears. Through XRD experiments, we can accurately determine the crystallinity of polysaccharide-coated SeNPs products (32). The XRD spectrum of DHP-SeNPs, as presented in Figure 6, reveals a broad peak at 2θ = 15° to 27°, and does not feature any sharp crystalline peaks. This observation indicates that the DHP-SeNPs generated through the redox reaction system possess an amorphous non-crystalline structure (33). This phenomenon is probably due to the adsorption of SeNPs onto DHPs-1 during their formation, which disrupts the crystallinity of selenium (17), consistent with previous publication (27). The diffraction peak range of DHPs-1 spans from 5° to 50°, whereas that of DHP-SeNPs extends from 5° to 65°. This finding further substantiates that SeNPs have effectively combined with DHPs-1, resulting in the formation of a new compound rather than merely a physical mixture of SeNPs and DHPs-1. Thus, UV absorption spectra, infrared absorption spectra, and transmission electron microscopy confirm that we have successfully synthesized DHP-SeNPs.

#### 3.1.8 Ultraviolet and infrared spectra

**Figure 7:**
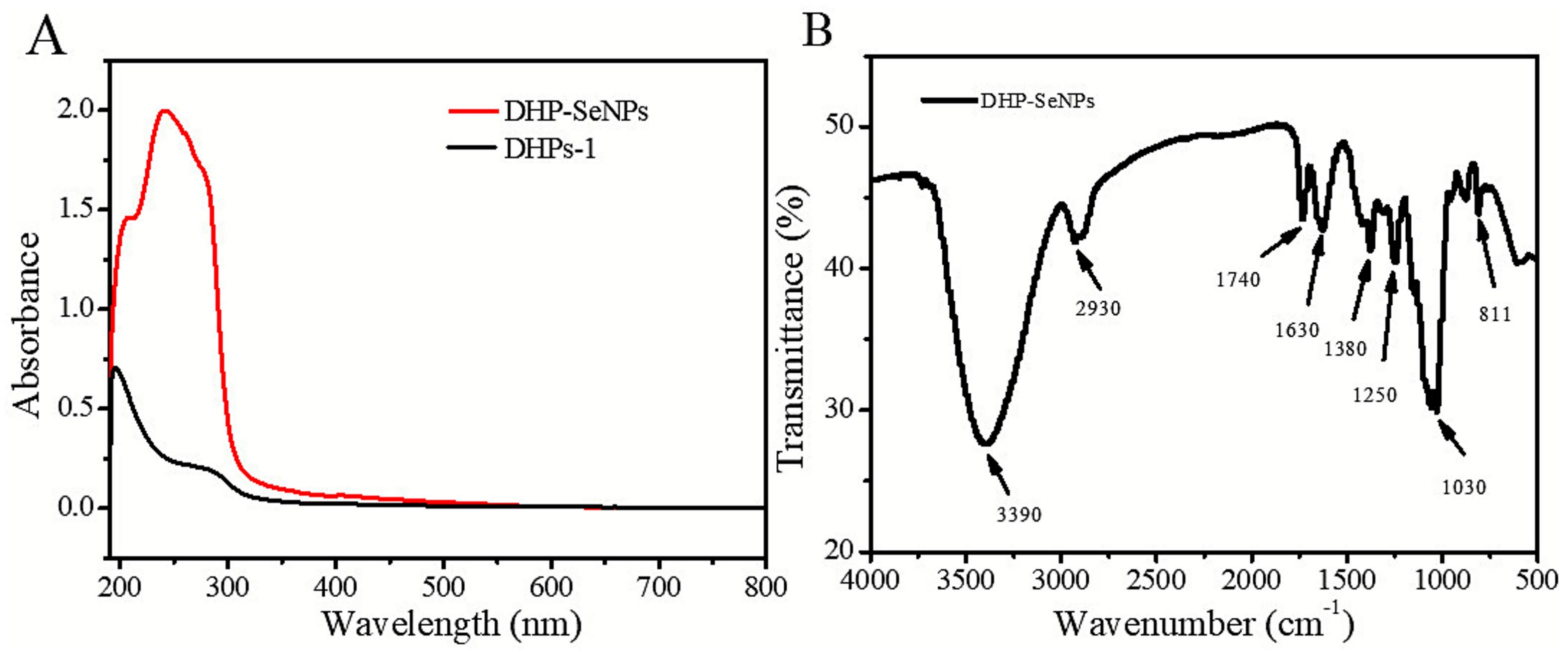
**(A)** Ultraviolet spectra of DHP-SeNPs; **(B)** Infrared spectra of DHP-SeNPs.

Figure 7A shows that the UV–Vis spectrum of DHP-SeNPs has a wide absorption band spanning from 200 to 600 nm, with the strongest peak at 242 nm. The phenomenon observed can be linked to the interaction of the O-H and C-O groups in DHPs-1 with the SeNPs surface. This interaction promotes the formation of new O-H-Se and C-O-Se bonds, leading to the development of DHP-SeNPs (34). This result is consistent with the UV spectral analysis reported by Liu et al. for selenium nanoparticles derived from goji berry polysaccharides (16). According to Figure 7B, the characteristic absorption of DHP-SeNPs was identified through FT-IR spectroscopy in the range of 500 to 4,000 cm^−1^. The absorption peak of DHP-SeNPs at approximately 3,390 cm^−1^ is linked to the stretching vibrations of O-H bonds found in or between carbohydrate molecules, a typical absorption peak for polysaccharides (35). This finding implies that the polysaccharide’s fundamental structure was not modified by the formation of DHP-SeNPs. As a result of hydrogen bonding interactions between the O-H groups of DHPs-1 and SeNPs, the hydroxyl peak of DHPs-1 was displaced from 3,423 cm^−1^ to 3,390 cm^−1^ (27). The stretching vibrations of C-H bonds are responsible for the weak absorption peak at 2930 cm^−1^, whereas the peak at 1380 cm^−1^ is due to the bending vibrations of C-H angles (4). The absorption peak at 1030 cm^−1^ is caused by C-O-H stretching and vibration of C-O-C bonds. This is the characteristic absorption of polysaccharides (36, 37). Infrared spectroscopy indicates that the polysaccharide structure of DHP-SeNPs remains largely unchanged after synthesis. This result suggests that intermolecular hydrogen bonding occurs between Se and the OH groups of DHPs-1, leading to the formation of Se-O bonds without disrupting the chemical bonds inherent in Se and OH groups. Consequently, this interaction results in DHPs-1-modified SeNPs with exhibiting a stable spherical structure (30, 38).

#### 3.1.9 Thermal properties

**Figure 8:**
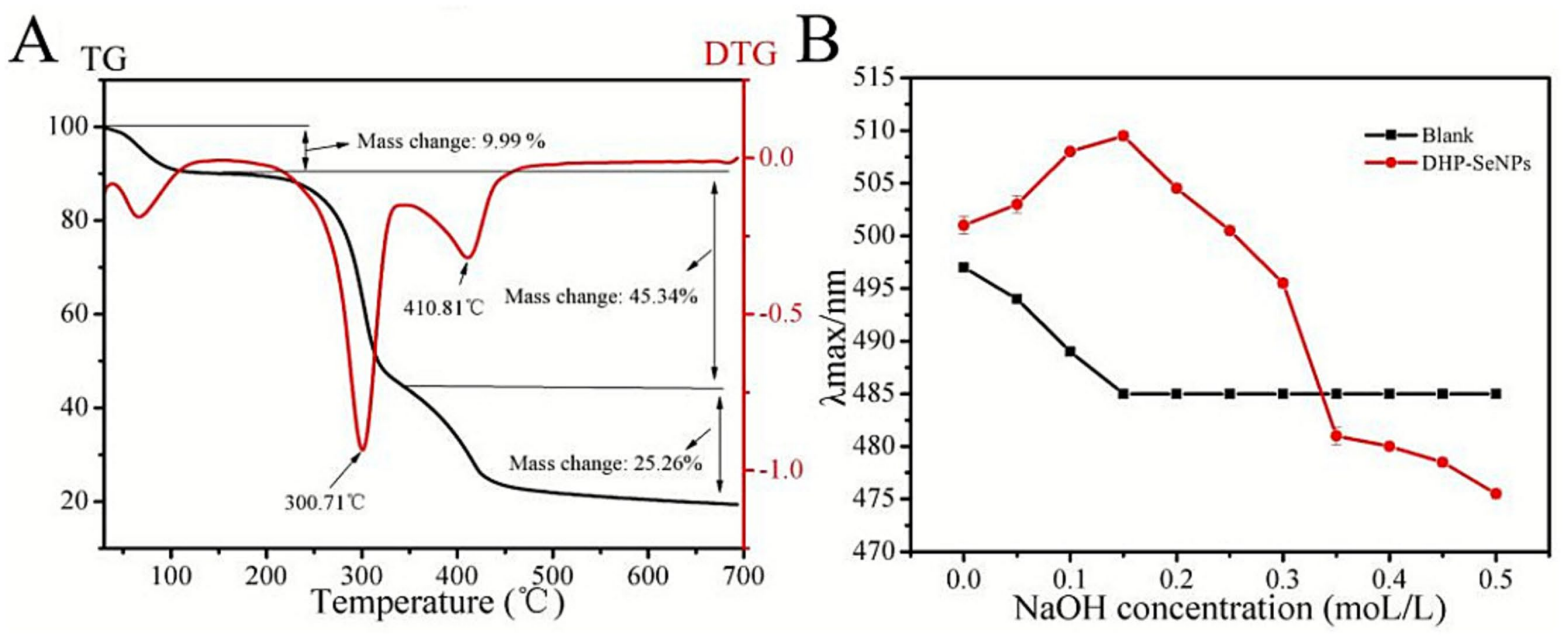
**(A)** TG and DTG curves of DHP-SeNPs; **(B)** the maximum absorption wavelengths of Congo red-DHP-SeNPs complexs and Congo red solution at various concentrations of NaOH.

When heated, DHP-SeNPs experience mass loss in three separate phases. As shown in Figure 8A, the first stage is between 30 to 155.43 °C, mainly due to free and bound water evaporation from DHP-SeNPs. The second stage of mass loss begins at 155.43 °C and concludes at 343.32 °C, with a total percentage of mass loss amounting to 45.34%. This significant reduction in mass is due to the cleavage of polysaccharide carbon chains and hydrogen bonds. The third stage occurs between 343.32 and 700 °C. During this phase, the rate of mass loss is relatively gradual, with an overall total mass loss of 25.26%, which can be ascribed to the slow pyrolysis of residual materials (35).

#### 3.1.10 Congo red test

Polysaccharides exhibit biological activities like anticancer effects, immune modulation, and antioxidant properties, which are closely associated with their triple helix conformation (39). Polysaccharides with a triple helical structure can form complexes with Congo red (40). Compared to the blank control, this complex experiences a red shift in its maximum absorption wavelength in the ultraviolet spectrum when placed in alkaline conditions (39). For this reason, the Congo red method is applied to evaluate the triple helical form of polysaccharides (41). From Figure 8B, it can be observed that within the range of 0 to 0.5 mol/L NaOH, the maximum absorption wavelength (λmax) of DHP-SeNPs initially increases and then decreases with increasing alkali concentration, while the control group shows a trend of initial decrease followed by stabilization. The results indicate a significant red shift in λmax at high alkaline concentrations (0.15 mol/L), suggesting the presence of a triple helix structure in DHP-SeNPs. Previous studies on DHPs-1 have shown that it exhibits only two stages of mass loss during heating (42). However, DHP-SeNPs have an additional third stage of mass loss, indicating that the thermal stability of DHPs-1 polysaccharides has changed after binding with selenium.

### 3.2 Analysis of antioxidant activity

#### 3.2.1 Reducing power assays

**Figure 9:**
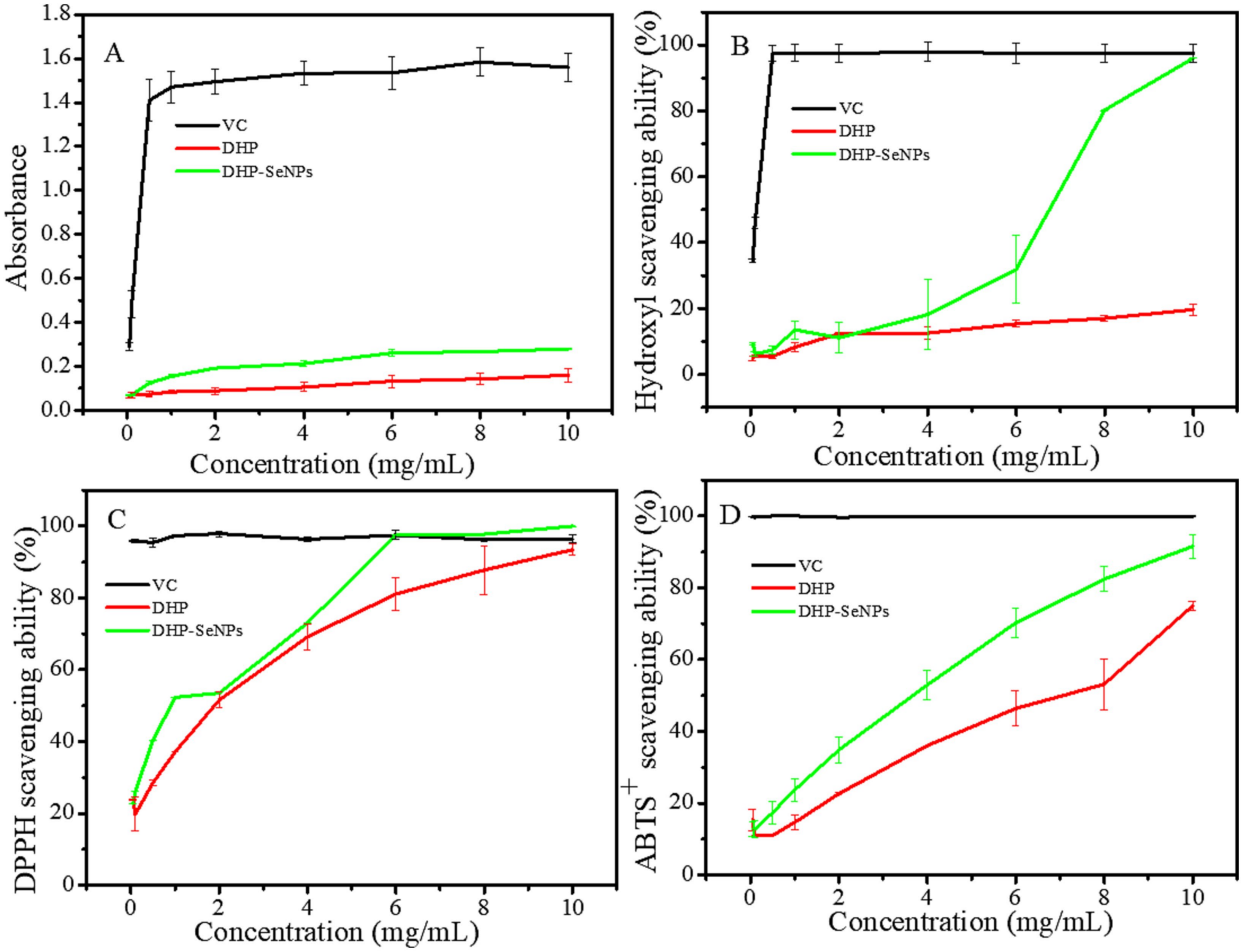
Antioxidant activity of DPHs-1 and DHP-SeNPs. **(A)** Reducing power; **(B)** hydroxyl radical scavenging activity; **(C)** DPPH radicals scavenging activity; **(D)** ABTS^+^ radical scavenging activity.

The ability of polysaccharides to transform trivalent iron ions into divalent iron ions is evaluated using the reducing power assay, which measures absorbance at a wavelength of 700 nm. Increased absorbance signifies greater reducing power, which is positively linked to antioxidant capacity. As shown in Figure 9A, DPHs-1 exhibits a positive correlation between its reducing ability and concentration within the range of 0.05 to 10 mg/mL. With increasing sample concentration, both the positive control Vc and DPHs-1 demonstrate enhanced reducing capabilities. At a concentration of 10 mg/mL, the absorbance of DPHs-1 at a wavelength of 700 nm was measured at 0.16. In comparison, the absorbance of DHP-SeNPs under identical conditions was found to be 0.29, indicating that its reducing power is significantly superior to that of DPHs-1. This indicates that combining SeNPs with DPHs-1 significantly enhances the antioxidant properties of DPH-1, and DHP-SeNPs demonstrates synergistic properties.

#### 3.2.2 Hydroxyl radical scavenging activity

Capable of crossing cell membranes, the hydroxyl radical is a very reactive species that interacts with biological molecules (42). The Fenton reaction produces hydroxyl radicals which react with salicylic acid to produce 2.3-di-hydroxybenzoic acid, with absorbance up to 510 nm. The addition of polysaccharides to the Fenton system can reduce the concentration of hydroxyl radicals, decrease the formation of colored substances, and lower the absorbance at 510 nm, thereby allowing for an assessment of their antioxidant capacity. In this experiment, Vc was used as a positive control to compare and analyze the ability of DHPs-1 and DHP-SeNPs to eliminate hydroxyl radicals at different concentrations. As shown in Figure 9B, DHPs-1 exhibited concentration-dependent hydroxyl radical scavenging activity within the range of 0.05 to 10 mg/mL, with the scavenging rate increasing from 5.06 to 19.76%. The concentration of DHP-SeNPs corresponding to a hydroxyl radical scavenging rate of 50% is approximately 6.9 mg/mL. At a concentration of 10 mg/mL, DHP-SeNPs demonstrated a hydroxyl radical scavenging rate approaching nearly 100%. These results indicate that the combination of SeNPs with DHPs-1 significantly enhances the antioxidant activity of DHPs-1 (43), suggesting that DHP-SeNPs have potential for development as natural antioxidants and functional food ingredients.

#### 3.2.3 DPPH free radical scavenging activity

In Figure 9C, it is shown that the scavenging activity of DHPs-1 becomes more pronounced as the concentration increases from 0.05 to 10 mg/mL. DHPs-1 reached a DPPH scavenging rate of 92.64% at a concentration of 10 mg/mL, similar to Vc. In contrast, polysaccharides extracted from *D. huoshanense* orchid using traditional water extraction methods exhibited lower scavenging rates within the same concentration range (44), indicating that the extraction and purification of DHPs-1 significantly influences its antioxidant activity. The DPPH scavenging rate of DHP-SeNPs also exhibited a concentration-dependent increase, surpassing that of DHPs-1 across the range of 0 to 10 mg/mL. The IC₅₀ values of DHP-SeNPs and DHPs-1 were 0.9 mg/mL and 1.9 mg/mL, respectively. When the concentration was 6 mg/mL, the DPPH scavenging rate of DHP-SeNPs was almost 100%, showing that SeNPs greatly improved the antioxidant capacity of DHPs-1. Scavenging these free radicals depended on the capacity to donate hydrogen, which could be boosted by the nano-effects and the synergistic interaction between polysaccharides and SeNPs (45).

#### 3.2.4 ABTS^+^ free radical scavenging activity

According to Figure 9D, the ABTS^+^ scavenging rates for DHPs-1 and DHP-SeNPs showed a consistent rise as the polysaccharide concentration increased from 0 to 10 mg/mL. At 10 mg/mL, DHPs-1 and DHP-SeNPs showed ABTS^+^ free radical scavenging activities of 74.95 and 92.75%. The IC₅₀ values of DHP-SeNPs and DHPs-1 are 3.8 mg/mL and 7.0 mg/mL, respectively. At identical concentrations, the scavenging rate of DHP-SeNPs surpassed that of DHPs-1, indicating that DHP-SeNPs possess a superior ability to scavenge ABTS^+^ compared to DHPs-1 alone. The presence of SeNPs and their size might be responsible for the improved clearance rate. Findings reveal that the heightened antioxidant activity of L-SeNP results from the modification of LPS, which enlarges the specific surface area and increases the availability of multiple reaction sites for free radicals (4). The antioxidant activity and free radical scavenging capability of SeNPs *in vitro* are also dependent on their size, according to relevant studies (46–48). Experimental results show that the smaller the DHP-SeNPs particles, the larger the specific surface, and therefore the larger the reaction point of the free radicals. In summary, both DHPs-1 and DHP-SeNPs demonstrate excellent ABTS^+^ scavenging capabilities within the tested range, highlighting their potential as novel antioxidants.

### 3.3 Analysis of immunomodulatory activity

#### 3.3.1 Cell viability

**Figure 10:**
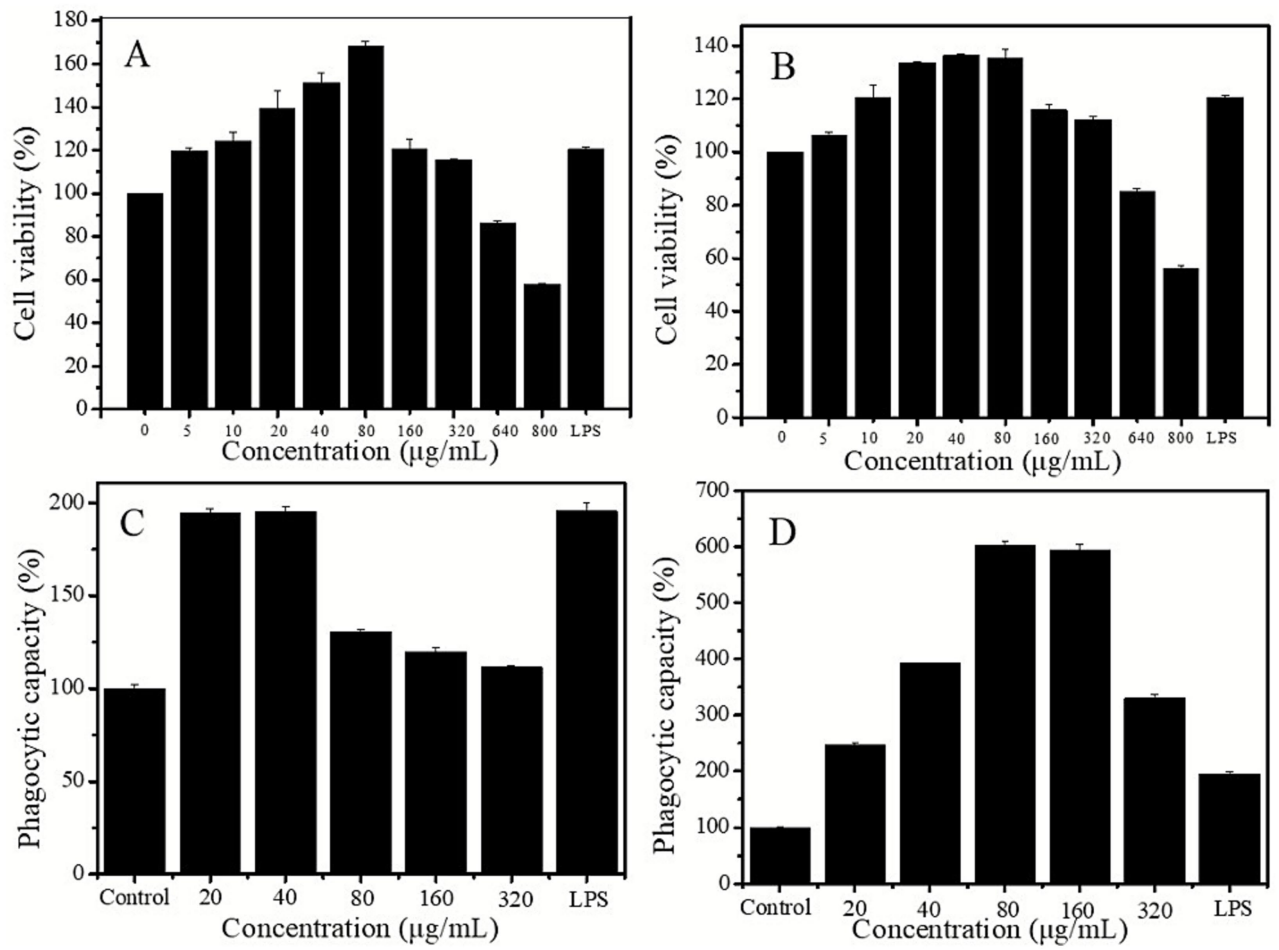
**(A,B)** Effects of DHPs-1 and DHP-SeNPs on cell viability of RAW264.7; **(C,D)** Effect of DHPs-1 and DHP-SeNPs on phagocytosis of RAW264.7 cells.

According to Figures 10A,B, both DHPs-1 and DHP-SeNPs were non-toxic to RAW264.7 cells at concentrations between 5 and 320 μg/mL, with all cells remaining alive. At a concentration of 80 μg/mL DHPs-1 demonstrated the highest proliferation activity compared to the control group, achieving a rate of 68%. However, at concentrations exceeding 320 μg/mL, DHPs-1 exhibited inhibitory effects on RAW264.7 cells, suggesting that DHPs-1 possesses cytotoxic properties within this concentration range. In comparing with existing studies (49), it was found that DHPs-1 has a higher macrophage proliferation rate. In summary, DHPs-1 can exert immunomodulatory effects by regulating the proliferation of RAW264.7 cells (50). As illustrated in Figure 10B, the suitable concentration of DHP-SeNPs for promoting cell proliferation is 40 μg/mL, which differs from that of DHPs-1. Based on these findings, a concentration range of 20–320 μg/mL is recommended for experimental treatments to assess the immunomodulatory effects of both DHPs-1 and DHP-SeNPs on RAW264.7 macrophages.

#### 3.3.2 Phagocytic capacity

Macrophages are highly conserved phagocytes in the process of continuous evolution. Phagocytosis is one of the most basic defense mechanisms (51). The CCK8 method was employed to detect the effects of DHPs-1 and DHP-SeNPs on the phagocytic activity of RAW264.7 cells, with results presented in in Figures 10C,D. Compared to the blank control group, all dosage groups of DHPs-1 and DHP-SeNPs showed increased absorption of neutral red by RAW264.7 macrophages, according to the results. Figure 10C demonstrates that within the concentration range of 20 to 320 μg/mL, DHPs-1 exhibited optimal performance at the low dosage group of 40 μg, with all experimental groups showing higher activity than the blank group but lower than the positive control group LPS. As depicted in Figure 10D, DHP-SeNPs displayed a strong ability to promote macrophage phagocytosis, with the optimal concentration being 80 μg/mL. From Figures 6C,D, it can be observed that at any identical concentration under the testing conditions, the capacity of DHP-SeNPs to enhance macrophage phagocytosis was superior to that of DHPs-1. It is indicated that DHP-SeNPs enhance the ability of macrophages to perform phagocytosis. Further investigation is necessary to uncover the underlying mechanism. In conclusion, both DHPs-1 and DHP-SeNPs are capable of boosting the phagocytic activity of RAW264.7 macrophages, thereby affecting immune activity, with DHP-SeNPs proving to be more effective.

#### 3.3.3 Cell scratch

**Figure 11:**
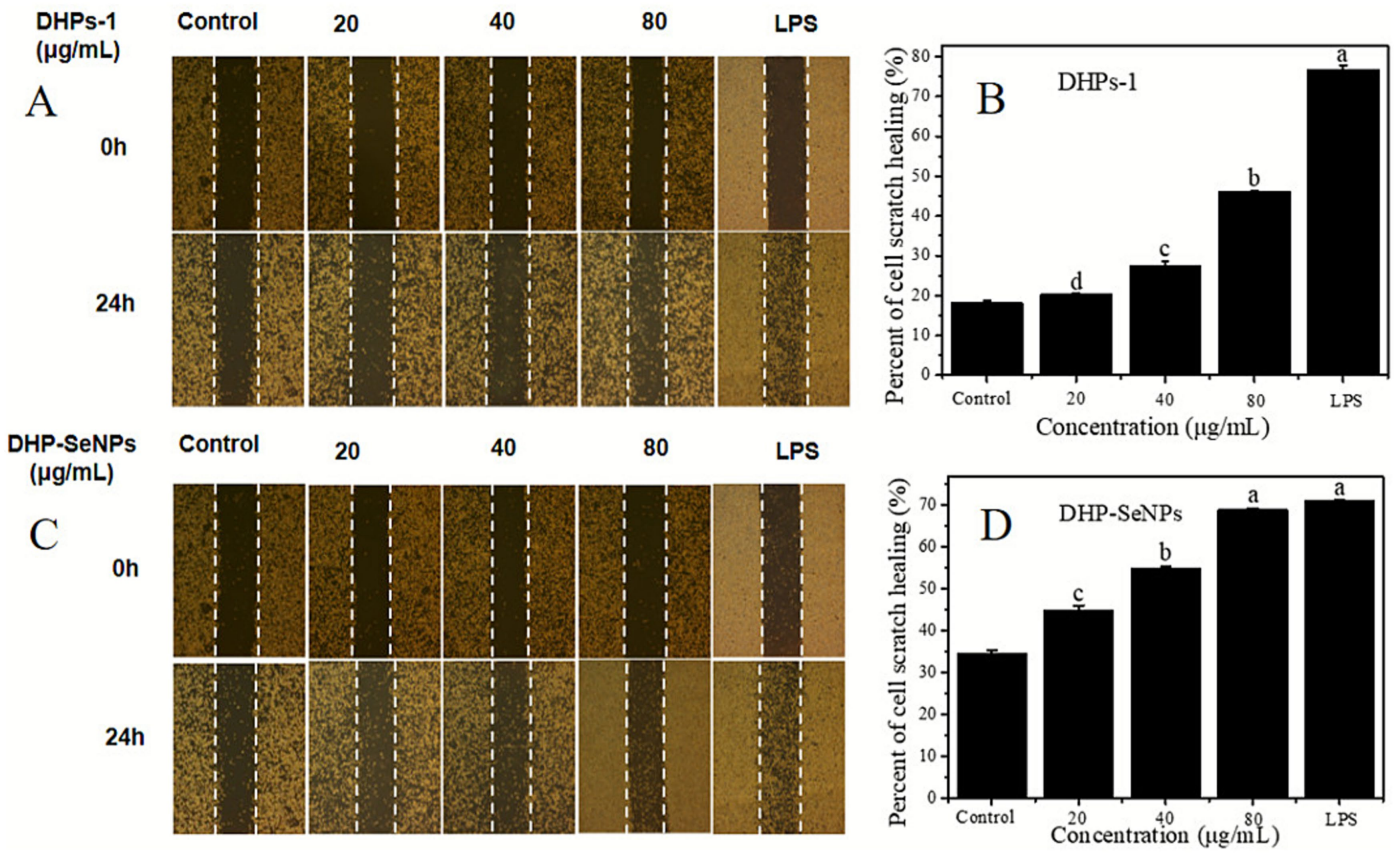
Results of cell scratch experiment: **(A,C)** Cell scratch image; **(B,D)** Percent of cell scratch healing.

As shown in Figures 11A–D, RAW264.7 macrophages were treated with different concentrations of DHPs-1, DHP-SeNPs (20 to 80 μg/mL), and LPS (1 μg/mL) to observe cell scratch images and calculate the cell migration rate. The results indicate that both DHPs-1 and DHP-SeNPs at concentrations of 20 to 80 μg/mL significantly promote the scratch healing of RAW264.7 macrophages compared to the control group. As seen in Figures 6G,H, at concentrations of 20, 40, and 80 μg/mL, DHP-SeNPs demonstrate a superior ability to promote the scratch healing of RAW264.7 macrophages compared to DHPs-1. This suggests that DHP-SeNPs can enhance the ability of DHPs-1 to promote macrophage scratch healing. These results indicate that both DHPs-1 and DHP-SeNPs can promote the migration of RAW264.7 cells to some extent, with DHP-SeNPs being more effective than DHPs-1.

#### 3.3.4 RT-PCR analysis of effects of DHPs-1 and DHP-SeNPs on the mRNA expression of TNF-*α*, IL-1β, IL-6, and IL-10

Cytokines such as IL-1β, IL-6, and TNF-α are important signaling molecules in the immune system. They activate immune cells in cells including T cells and macrophages and trigger immune responses. The anti-inflammatory cytokine IL-10 can inhibit the overactivation of macrophages, and through its regulatory mechanisms, it can prevent the harmful effects associated with the overactivation of immune responses (52).

##### 3.3.4.1 Effects of DHPs-1 and DHP-SeNPs on TNF-α mRNA expression

**Figure 12:**
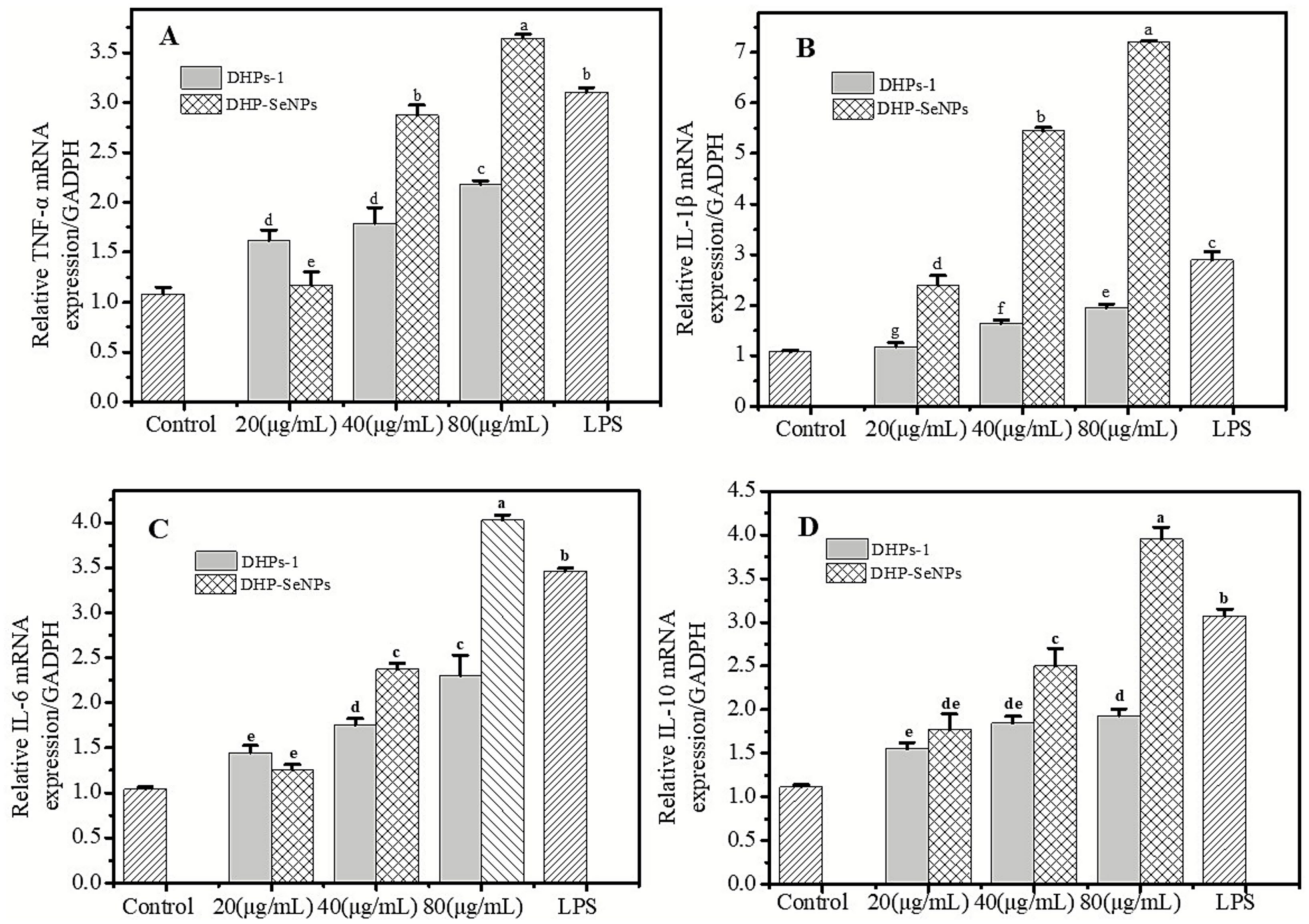
Effects of DHPs-1 and DHP-SeNPs on **(A)** TNF-*α*; **(B)** IL-1β; (B) **(C)** IL-6 and **(D)** IL-10 mRNA expression in RAW264.7 macrophages.

TNF-α plays a vital role in immunoregulation by activating macrophages, increasing their functional responses, and inducing the production of immune and inflammatory mediators. Figure 12A illustrates that DHPs-1 and DHP-SeNPs, at concentrations between 20 and 80 μg/mL, enhance TNF-α mRNA expression in RAW264.7 macrophages compared to the blank control group, showing a dose-dependent pattern. At concentrations of 40 μg/mL and 80 μg/mL, DHP-SeNPs significantly outperformed DHPs-1 in promoting the mRNA expression of TNF-α in macrophages (*p* < 0.05). At a concentration of 20 μg/mL, DHP-SeNPs have a slightly lesser promoting effect compared to DHPs-1, though the difference is not significant. The activity of DHP-SeNPs is significantly higher than that of DHPS-1 at concentrations of 40 μg/mL and 80 μg/mL (*p* < 0.05). At 80 μg/mL, the effect of DHP-SeNPs surpassed that of the positive control group LPS (1 μg/mL), indicating that the polysaccharide nano-selenium from *D. huoshanense* has a superior capacity to promote TNF-α mRNA expression compared to DHPs-1. This further suggests that DHP-SeNPs have the potential to enhance immune activity.

##### 3.3.4.2 Effects of DHPs-1 and DHP-SeNPs on the expression of IL-1β and IL-6 mRNA

TNF-α can modulate the cytokine cascade response, leading to the release of Interleukin-1β (IL-1β) and Interleukin 6 (IL-6). Leukocytes release interleukins, which act as proteins and signaling molecules. They facilitate the maturation and growth of primitive cells derived from bone marrow and regulate the body’s immune defense mechanisms. IL-1β enhances immunity by activating B lymphocytes and by activating NK cells to increase their cytotoxic efficacy. As indicated in Figure 12B, both DHPs-1 and DHP-SeNPs can boost the mRNA expression of IL-1β in macrophages within the 20 to 80 μg/mL concentration range, with DHP-SeNPs exhibiting a significantly stronger effect than DHPs-1. The activity of both DHPs-1 and DHP-SeNPs increases with increasing concentration. DHP-SeNPs exhibit a robust capacity to control IL-1β mRNA expression in macrophages. At 40 μg/mL, they are 3.32 times as effective as DHPs-1, and at 80 μg/mL, with effects 3.7 times that of DHPs-1. The significant enhancement of DHPs-1 activity through SeNPs binding could be responsible for the potent immunomodulatory activity of DHP-SeNPs. IL-6, a cytokine with various roles, is significant in immune responses (53). Figure 12C illustrates that both DHPs-1 and DHP-SeNPs enhance IL-6 mRNA expression in macrophages at concentrations between 20 and 80 μg/mL, with the effect becoming more pronounced as the concentration increases. The activity of DHP-SeNPs is significantly higher than that of DHPs-1 at concentrations of 40 μg/mL and 80 μg/mL (*p* < 0.05). Notably, at a concentration of 80 μg/mL, the mRNA expression of IL-6, which promotes macrophage activity, is 1.75 times greater in the presence of DHP-SeNPs compared to DHPs-1. The observed results show that SeNPs binding to DHPs-1 significantly boosts the promoting effect of DHPs-1 on IL-6 mRNA expression in macrophages, with the underlying mechanism needing more detailed study.

##### 3.3.4.3 Effects of DHPs-1 and DHP-SeNPs on IL-10 mRNA expression

Excessive secretion of pro-inflammatory factors may exacerbate inflammation at the site, thereby hindering the repair of damaged cells and tissues. Anti-inflammatory cytokines such as IL-10 can be utilized to prevent the harmful effects of excessive macrophage activation. IL-10 is an important anti-inflammatory cytokine that has the effect of inhibiting the immune system (54–56). Figure 12D illustrates that both DHPs-1 and DHP-SeNPs enhance IL-10 mRNA expression in macrophages at concentrations between 20 and 80 μg/mL. However, at 40 μg/mL and 80 μg/mL, DHP-SeNPs significantly outperformed DHPs-1. At 80 μg/mL, DHP-SeNPs demonstrated activity that was significantly superior to the positive control group LPS (1 μg/mL) (*p* < 0.05). This indicates that both DHPs-1 and DHP-SeNPs can regulate the mRNA expression of the anti-inflammatory factor IL-10 in macrophages, effectively preventing the harmful effects caused by the excessive secretion of pro-inflammatory factors (52).

## 4 Conclusion

The present work reported the preparation and characterization of a DPHs-1 from *D. huoshanense* and its influence on bioactivities of DPH-SeNPs. This study systematically investigates the binding characteristics and properties of DHPs-1 with SeNPs using multiple characterization techniques. UV and infrared spectroscopy confirmed that the formation of DHP-SeNPs did not disrupt the functional group structure of the polysaccharide. Rather, spherical SeNPs were associated with DHPs-1 through the formation of Se-O bonds, resulting in the creation of new chelates. SEM revealed that the surface of DHPs-1 became rough after binding with SeNPs, while TEM confirmed that DHP-SeNPs are smooth, uniformly dispersed spherical particles with an average particle size of 61 nm. XRD analysis indicated that the diffraction peak range changed, leading to the formation of a new substance. FT-IR spectroscopy confirmed that SeNPs are stably bound to DHPs-1 through Se-O bonds. Congo red test showed that the formation of selenium nanoparticles did not disrupt the triple helix structure of DHPs-1, and thermal analysis demonstrated that DHP-SeNPs exhibit high thermal stability under physiological conditions. These results indicate that the binding mode of DHPs-1 with SeNPs is unique, and the formed nanoparticles exhibit good stability and potential application value.

In the study of the *in vitro* antioxidant activity of DHPs-1 and DHP-SeNPs composites, DHP-SeNPs exhibited enhanced reducing power. At a concentration of 10 mg/mL, the scavenging rate of DHP-SeNPs against hydroxyl radicals approaches 100%, whereas the scavenging rate of DHPs-1 is approximately 20%. In the experiments involving the scavenging of DPPH and ABTS^+^ radicals, the activity of DHP-SeNPs also surpassed that of DHPs-1, with both activities increasing with sample concentration. The experiments demonstrated that DHPs-1 adsorbed onto SeNPs significantly enhanced the in vitro antioxidant activity of DHPs-1, providing potential applications for DHP-SeNPs as natural polysaccharide antioxidants and selenium supplements. Cell experiments results showed that within an appropriate concentration range, both DHPs-1 and DHP-SeNPs exhibited no toxicity to RAW264.7 cells, and could significantly promote the phagocytic and migratory capabilities of RAW264.7 macrophages. Meanwhile, they could regulate the mRNA expression levels of TNF-*α*, IL-1β, IL-6 and IL-10, thereby balancing cellular immune activity and avoiding the adverse effects caused by excessive activation of macrophages. The results of this study provide a theoretical basis for the application of DHP-SeNPs in selenium supplementation, antioxidant activity, and immune function enhancement in the food and pharmaceutical fields. In addition, future studies should perform comprehensive safety assessments of DHP-SeNPs via toxicity and hemocompatibility testing, alongside mechanistic explorations.

## Data Availability

The original contributions presented in the study are included in the article/supplementary material, further inquiries can be directed to the corresponding authors.
